# Dietary Curcumin Attenuates Hepatic Cellular Senescence by Suppressing the MAPK/NF-κB Signaling Pathway in Aged Mice

**DOI:** 10.3390/antiox12061165

**Published:** 2023-05-27

**Authors:** Da-Yeon Lee, Su-Jeong Lee, Prabha Chandrasekaran, Gopal Lamichhane, Jennifer F. O’Connell, Josephine M. Egan, Yoo Kim

**Affiliations:** 1 Department of Nutritional Sciences, Oklahoma State University, Stillwater, OK 74078, USA; dayeon.lee@okstate.edu (D.-Y.L.); crystal.lee10@okstate.edu (S.-J.L.);; 2 Laboratory of Clinical Investigation, National Institute on Aging, Baltimore, MD 21224, USA; prabha.chandrasekaran@nih.gov (P.C.); jennifer.oconnell@nih.gov (J.F.O.); eganj@grc.nia.nih.gov (J.M.E.)

**Keywords:** curcumin, liver, cellular senescence, MAPK, NF-κB, senolytic

## Abstract

Dietary interventions with bioactive compounds have been found to suppress the accumulation of senescent cells and senescence-associated secretory phenotypes (SASPs). One such compound, curcumin (CUR), has beneficial health and biological effects, including antioxidant and anti-inflammatory properties, but its ability to prevent hepatic cellular senescence is unclear. The objective of this study was to investigate the effects of dietary CUR as an antioxidant on hepatic cellular senescence and determine its benefits on aged mice. We screened the hepatic transcriptome and found that CUR supplementation led to the downregulation of senescence-associated hepatic gene expressions in both usually fed and nutritionally challenged aged mice. Our results showed that CUR supplementation enhanced antioxidant properties and suppressed mitogen-activated protein kinase (MAPK) signaling cascades in the liver, particularly c-Jun N-terminal kinase (JNK) in aged mice and p38 in diet-induced obese aged mice. Furthermore, dietary CUR decreased the phosphorylation of nuclear factor-κB (NF-κB), a downstream transcription factor of JNK and p38, and inhibited the mRNA expression of proinflammatory cytokines and SASPs. The potency of CUR administration was demonstrated in aged mice via enhanced insulin homeostasis along with declined body weight. Taken together, these results suggest that CUR supplementation may be a nutritional strategy to prevent hepatic cellular senescence.

## 1. Introduction

Cellular senescence, defined as a permanent cell cycle arrest characterized by an exponential accumulation of cellular damage in aged tissues, appears to be one of the important hallmarks of aging and age-related diseases, including obesity, type 2 diabetes mellitus, cardiovascular disease, and cancer [1,2,3,4,5]. Senescent cells secrete proinflammatory cytokines and chemokines, known as senescence-associated secretory phenotypes (SASPs), which can alter cellular structure and functions, inhibit the proper functioning of the immune system, and subsequently cause low-grade systemic inflammation called ‘inflammaging’ [6,7]. Previous studies have suggested that mitogen-activated protein kinase (MAPK) pathways play pivotal roles in the development of senescence traits via suppressing cellular growth, increasing apoptosis resistance, and stimulating SASP production [8,9]. Therefore, eliminating senescent cells is a promising therapeutic strategy to attenuate or prevent aging and age-associated diseases.

Consistent with these approaches, researchers have introduced some drugs and chemicals called ‘senolytics’ which induce selective clearance of senescent cells via apoptosis [10]. Multiple pieces of evidence have elucidated that senolytic compounds can improve physiological functions and extend health span, as well as ameliorate various age-related chronic disorders in aged animal models [11,12,13,14,15]. Yet, since some synthetic drugs exhibited mild or significant side effects in several in vitro and in vivo studies, phytochemicals from natural food resources can be alternatives to target senescent cells with low or no cytotoxicity [16,17]. One of the candidates of non-toxic senolytics is curcumin (CUR), a polyphenol that represents about 2–8% of the rhizome of the herbaceous perennial spice *Curcuma longa* L. (turmeric) [18,19]. The pharmacological benefits of curcumin have widely been described regarding its antioxidant, anti-inflammatory, anti-obesity, and anti-diabetic properties, suggesting its potential roles in mediating age-associated diseases [20,21,22]. However, whether curcumin can delay or prevent hepatic cellular senescence in aged in vivo models is not fully elucidated yet.

Based on this conceptualization, we hypothesized that dietary CUR may provide a senolytic effect by regulating senescence pathways in the liver through its pleiotropic traits. Thus, the purpose of the current study was to investigate the protective role of curcumin supplementation in hepatic cellular senescence and discover its health benefits in naturally aged mice.

## 2. Materials and Methods

### 2.1. Animals

All animals were housed in an American Association for Accreditation of Laboratory Animal Care (AAALAC)-accredited facility, and the procedures were approved by the Animal Care and Use Committee of the National Institute on Aging (NIA) and the Institutional Animal Care and Use Committee (IACUC) at Oklahoma State University. Aged male C57BL/6 mice (18–20 months old) were obtained from the NIA Aged Rodent Colony housed at the Charles River Laboratories (Frederick, MD, USA, or Raleigh, NC, USA). After being transferred into the NIA intramural housing facility (Baltimore, MD, USA) or the Oklahoma State University animal facility (Stillwater, OK, USA), the animals were acclimated for 1 week with a standard NIH chow diet (Teklad Global Rodent Diet; Envigo, Indianapolis, IN, USA). The mice were subjected to body weight and body composition measurements for baseline assessment, and they were then randomized into four groups: a normal chow diet (NCD; *n* = 7–9), an NCD with 0.4% (*w*/*w*) curcumin (NCD+CUR; *n* = 7–9), a high-fat high-sugar diet (HFHSD; *n* = 8), or an HFHSD with 0.4% (*w*/*w*) curcumin (HFHSD+CUR; *n* = 7) for 6 and 15 weeks (total of 16 to 18 mice per group in both studies). CUR, purchased from Sigma-Aldrich (St. Louis, MO, USA), was used for the customized diet formulation by Dyets Inc. (Bethlehem, PA, USA) as well as other diets. The dosage of CUR was determined according to previous studies using middle-aged mice, which was equivalent to 2 g/day for a 60 kg adult calculated with an equivalent surface area dosage conversion method [11,23,24,25]. The mice were allowed ad libitum access to food and water during the study. Their body weight and food consumption were monitored every week for both the 6- and 15-week intervention studies.

### 2.2. Glucose and Insulin Tolerance Test

The glucose tolerance test (GTT) was performed by measuring blood glucose levels from the tail veins of 16 h-fasted mice at 0 min and at 15, 30, 60, 90, and 120 min after intraperitoneal injection of 1 g/kg of glucose made with 50% (*w*/*w*) glucose solution (Alpha Teknova Inc., Hollister, CA, USA). For the insulin tolerance test (ITT), blood glucose levels were measured from the tail veins of 6 h-fasted mice at 0 min and at 15, 30, 60, 90, and 120 min following intraperitoneal administration of recombinant human insulin (0.75 U/kg body weight; Novo Nordisk Inc., Plainsboro, NJ, USA). Blood glucose levels were measured using a hand-held glucometer (CONTOUR^®^ NEXT EZ; Ascensia Diabetes Care, Parsippany, NJ, USA).

### 2.3. Senescence-Related Gene Profiling Analyses

Differentially expressed genes (DEGs) were identified using the RNA sequencing method as described in Lee et al. [11]. In brief, the library of total RNA from the liver was prepared using an Illumina TruSeq Stranded mRNA Kit, and sequencing was performed on a NovaSeq 6000 Sequencing System (Illumina, Inc., San Diego, CA, USA) with 2 × 100 bp read length. The reads were mapped to mm10 genome of Mus musculus (C57BL/6 strain), and the aligned results were added to Cuffdiff to identify DEGs. The associated Gene Expression Omnibus (GEO) accession number deposited to NCBI is GSE186971. The cellular senescence Gene Ontology (GO) analysis was performed by obtaining the gene list from the Mouse Genome Informatics (MGI) database (GO:0090398; http://www.informatics.jax.org) accessed on 28 October 2022 and the pathway analysis from the Kyoto Encyclopedia of Genes and Genomes (KEGG) database gene list (Pathway: hsa04218; https://www.genome.jp/kegg/pathway.html) accessed on 8 December 2022. Genes exhibiting *p*-value < 0.05 were considered significant. The data analyses and visualizations were performed using R Studio 1.4.1106 (http://www.R-project.org) accessed on 8 December 2022, and the figures were assembled in Adobe Photoshop (ver. 24.5).

### 2.4. Mouse Primary Hepatocyte Isolation and Treatments

Primary hepatocytes from 18-month-old female C57BL/6 mice were isolated using a two-step collagenase perfusion method. In brief, the mice were euthanized using a keta-mine/xylazine cocktail, and then their livers were rapidly perfused in situ via the portal vein using a perfusion buffer (Ca^2+^-free 1X Krebs Ringer HEPES, 0.5 M EGTA, pH of 7.4), for 4 min at 42 °C. After that, the livers were digested by a Ca^2+^/Mg^2+^-free perfusion buffer (1× CaCl_2_, without EGTA, pH 7.4) containing type I collagenase (from *Clostridium histolyticum*; Sigma-Aldrich) for 7 min. The digested liver tissues were collected, mechanically isolated, and purified using density-based separation with 30% Percoll (Sigma-Aldrich) gradient centrifugation. After culturing and incubating for 24 h, primary hepatocytes were treated with 10 μM of CUR or 100 μM of palmitic acid (PA; Cayman Chemical, Ann Arbor, MI, USA) for 24 h. Dimethyl sulfoxide (DMSO; Sig-ma-Aldrich) and bovine serum albumin (BSA; Cayman Chemical) were used as the controls, respectively.

### 2.5. Immunoblotting Analyses

The mouse liver tissues and primary hepatocytes were homogenized in T-PER™ Tissue Protein Extraction Reagent and RIPA Lysis and Extraction Buffer (Thermo Fisher Scientific, Waltham, MA, USA), respectively, as well as in PhosSTOP™ phosphatase inhibitor and cOmplete™ Mini Protease Inhibitor Cocktail (Sigma-Aldrich) via the OMNI BeadRuptor 24 (Omni-Inc., Kennesaw, GA, USA). To isolate nuclear and cytoplasmic fractions, NE-PER Nuclear and Cytoplasmic Extraction Reagents were used following the manufacturer’s instructions (Thermo Fisher Scientific). Protein concentration was measured using a bicinchoninic acid (BCA) assay kit (Thermo Fisher Scientific), and then, the protein loading samples were resolved in SDS-PAGE under reducing conditions and transferred to polyvinylidene fluoride (PVDF) membranes. The membranes were blocked in a blocking reagent (LI-COR, Lincoln, NE, USA) at room temperature for 1 h and incubated with the primary antibodies at 4 °C overnight as follows: phosphorylated extracellular signal-regulated kinase (p-ERK) 1/2, ERK1/2, phosphorylated c-Jun N-terminal kinase (p-JNK) 1/2, JNK1/2, p-p38, p38, phosphorylated MAP kinase-activated protein kinase 2 (p-MK2), MK2, p-p65, p65, p53, cyclophilin B, histone deacetylase 1 (HDAC1), β-tubulin, and β-actin from Cell Signaling Technology (Danvers, MA, USA). The membranes were washed with tris-based saline with Tween 20 (TBS-T), and the appropriate secondary antibodies (anti-rabbit IgG from Cell Signaling Technology or anti-mouse IgG from Santa Cruz Biotechnology, Inc., Dallas, TX, USA) were added in 5% non-fat dry milk for 1 h at room temperature. The membranes were washed with TBS-T three times and developed using a chemiluminescence assay system (Thermo Fisher Scientific). The bands on the membranes were visualized on autoradiographic X-ray films (Thomas Scientific, Swedesboro, NJ, USA). The Western blot images were scanned, saved as Tiff files, and inverted, and the integrated density was analyzed using ImageJ software (NIH). Phosphorylated protein levels were normalized to their respective total protein levels.

### 2.6. Real-Time Reverse-Transcription Polymerase Chain Reaction (RT-PCR)

Total RNA was extracted from the frozen tissue samples, and primary hepatocytes were harvested using TRIzol Reagent (Invitrogen; Thermo Fisher Scientific) and quantified using a NanoDrop OneC Microvolume UV-Vis Spectrophotometer (Thermo Fisher Scientific). The normalized RNA was synthesized into cDNA with an iScript™ cDNA Synthesis Kit (Bio-Rad Laboratories, Inc., Hercules, CA, USA). Gene expression was assessed using SYBR^®^ Green PCR Master Mix (Applied Biosystems; Thermo Fisher Scientific) on a CFX Opus 384 Real-Time PCR System (Bio-Rad Laboratories, Inc) through the following thermal cycling conditions: 95 °C for 10 min, followed by 39 cycles at 95 °C for 15 sec and 60 °C for 1 min. The fluorescence cycle threshold value (Ct) data were normalized to 18S ribosomal RNA and β-actin. The primer sequences are described in Table S1.

### 2.7. Statistical Analyses

All data were analyzed using GraphPad Prism 9 (GraphPad Software, San Diego, CA, USA). Ordinary two-way repeated-measure analysis of variance (ANOVA) was performed to analyze body weights, cumulative food intakes, food efficiency, blood glucose levels, GTT, and ITT, followed by Tukey’s multiple comparison tests. Quantitative data are represented as the mean ± standard error of the mean (SEM). The quantification analyses for the area under the curve (AUC), Western blot band density, and mRNA expressions were conducted using one-way ANOVA, followed by Tukey’s multiple comparison test after the outlier test (α = 0.05).

## 3. Results

### 3.1. Dietary Curcumin Alters Hepatic Senescence-Related Gene Profiling in Aged Mice

**Figure 1:**
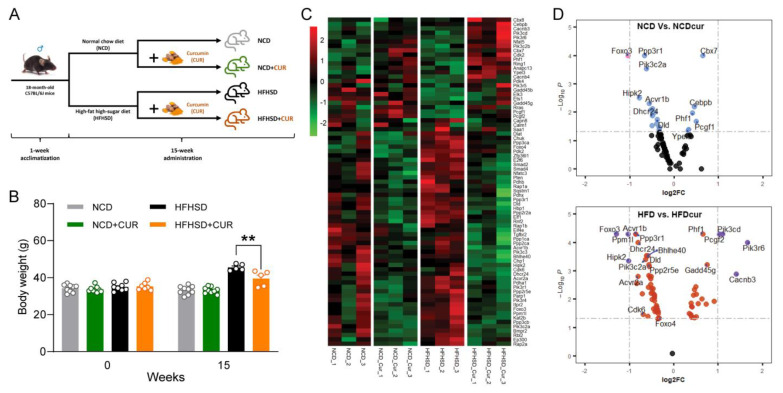
Curcumin attenuates obesity and alters hepatic senescence-related gene expression in HFHSD-induced obese aged mice. (**A**) 15-week animal experimental design. (**B**) body weight (g) measured at baseline and after 15 weeks of diet (*n* = 7–9 per group) (** *p* < 0.01). (**C**) Heatmap of senescence genes’ RNA expression as measured using FPKM (FPKM > 1.5). The list of genes was combined from the Cellular Senescence Gene Ontology (GO:0090398; MGI) and the KEGG pathway (hsa04218). Only expressions of genes that show significant differences in the NCD-Cur group compared to the NCD group or in the HFHSD-Cur group compared to the HFHSD group are presented in the heatmap (*p* < 0.05). Red indicates a positive Z-Score and green indicates a negative Z-score. (**D**) Volcano plots showing the log2 fold-change gene expression and associated significance (−Log_10_P) for the NCD-Cur to NCD comparison (upper) and the HFHSD-Cur to HFHSD comparison (lower). NCD, normal control diet; HFHSD, high-fat high-sugar diet; Cur, curcumin.

Using the experimental design of dietary CUR intervention for 15 weeks in the current study (Figure 1A), the comparison of body weights among the four groups measured at weeks 0 and 15 are shown in Figure 1B. We observed that the average body weight gain of the HFHSD+CUR group (39.57 ± 1.88 g) was remarkably lower than that of the HFHSD group (45.58 ± 0.77 g) after 15 weeks of intervention (*p* < 0.01), while there was no significant difference between the NCD and NCD+CUR groups at week 15 (Figure 1B).

We previously reported that dietary CUR supplementation altered the hepatic gene profiling in aged mice compared with non-supplemented mice [11]. To evaluate if CUR supplementation is associated with senescence, we performed a secondary analysis of our transcriptomic data [11]. The heatmap analysis of all genes associated with the senescence GO terms (Figure S1A) and KEGG pathways (Figure S1B) revealed the downregulation of a large proportion of HFHSD-driven genes with CUR supplementation (green as being downregulated, and red as being upregulated). Of the 157 genes in the combined gene list from the senescence GO and pathway analysis, 75 genes showed significant differences (*p* < 0.05) in the CUR-supplemented mice compared to their counterparts. The heatmap shows that CUR-supplemented mice downregulate overall gene expressions in the senescence pathways (Figure 1C). The log2 fold-change expressions are displayed as the volcano plots of two different dietary regimes, NCD (Figure 1D, upper) and HFHSD (Figure 1D, lower). The plot demonstrates that the CUR supplementation-driven changes in senescence gene expressions are more pronounced in the HFHSD group. It is highlighted that CUR supplementation contributes to the suppression of senescence genes in aging, and this phenomenon is more obvious in the combination of aging and obesity.

### 3.2. Dietary Curcumin Expresses Hepatic Antioxidant Properties in Aged Mice

**Figure 2:**
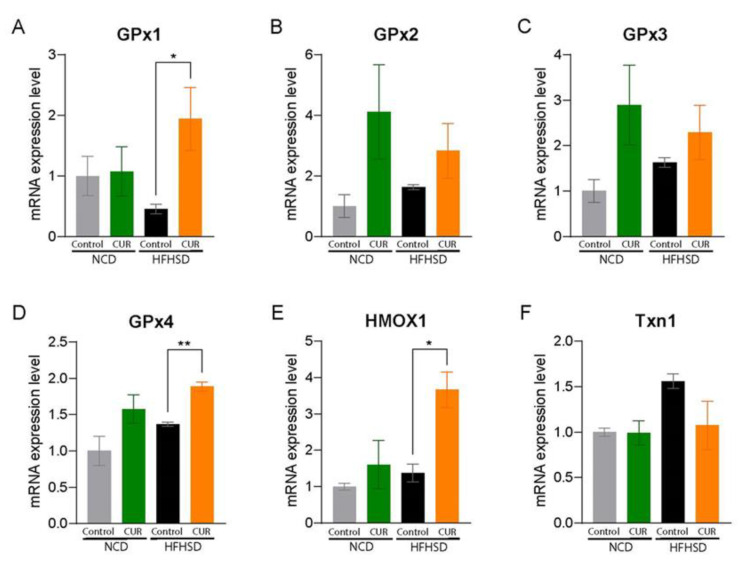
Curcumin expresses hepatic antioxidant properties in whole liver tissues from aged mice. mRNA expression levels of antioxidant enzymes: (**A**) GPx1, (**B**) GPx2, (**C**) GPx3, (**D**) GPx4, (**E**) HMOX1, and (**F**) Txn1. The results are expressed as mean ± SEM (* *p* < 0.05, ** *p* < 0.01).

**Figure 3:**
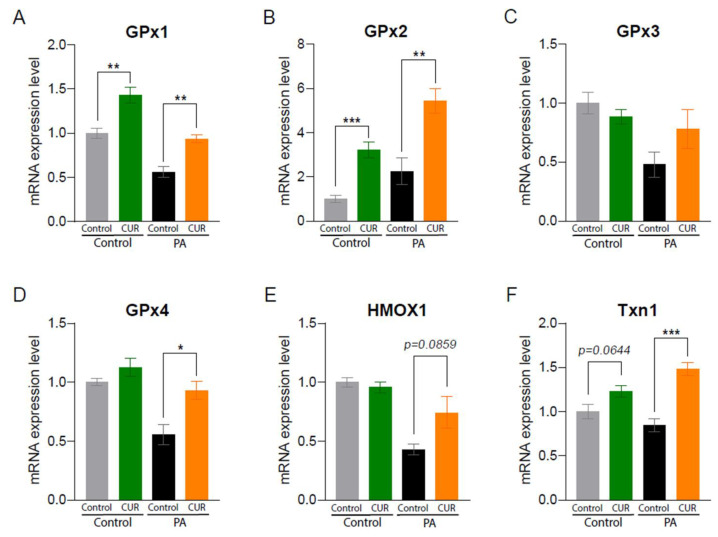
Curcumin expresses hepatic antioxidant properties in primary mouse hepatocytes. We isolated primary hepatocytes from 18-month-old female C57BL/6 mice. Hepatocytes were treated with fatty acid-free bovine serum albumin (BSA)-conjugated palmitic acid (PA, 100 μM of final concentration) mimicking HFHSD or with BSA as an NCD control with or without 10 μM of CUR treatment for 24 h. mRNA expression levels of antioxidant enzymes: (**A**) GPx1, (**B**) GPx2, (**C**) GPx3, (**D**) GPx4, (**E**) HMOX1, and (**F**) Txn1. The results are expressed as mean ± SEM (* *p* < 0.05, ** *p* < 0.01, *** *p* < 0.005).

The strong antioxidant capacity of CUR has been well established for decades through improving metabolic abnormalities in multiple metabolic organs [26]. We further assessed gene expressions related to oxidative stress and antioxidant enzymes in whole liver tissue samples and mouse primary hepatocytes. CUR supplementation significantly increased the mRNA expressions of glutathione peroxidase (GPx) 1, GPx4, and heme oxygenase 1 (HMOX1) in the HFHSD+CUR group compared to the HFHSD group (Figure 2). While CUR administration slightly improved the antioxidant gene expressions in the NCD group, there was no significance (Figure 2). To confirm these findings, we isolated primary hepatocytes, the parenchymal cells in charge of various metabolic mechanisms, and treated them with CUR under normal and obesogenic environments. Consistent with the in vivo results, CUR supplementation significantly upregulated the expressions of GPx1, GPx2, GPx4, and Txn1 in the BSA-conjugated palmitic acid (PA)-treated primary hepatocytes (Figure 3A,B,D,F), whereas the expression of GPx1 was remarkably enhanced by CUR in the BSA-treated primary hepatocytes (Figure 3A).

### 3.3. Dietary Curcumin Suppresses MAPK Signaling Pathways in Aged Mice

**Figure 4:**
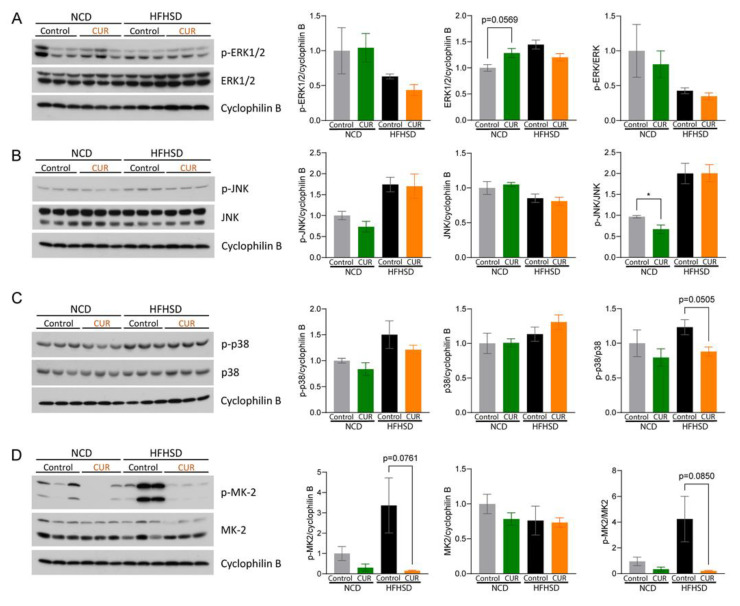
Curcumin ameliorates hepatic cellular senescence by regulating the p38-MAPK signaling pathway in HFHSD-fed aged mice. Immunoblots and protein expression levels in the liver tissue lysates after 15 weeks of dietary interventions: (**A**) p-ERK1/2, ERK1/2, and p-ERK/ERK ratio; (**B**) p-JNK, JNK, and p-JNK/JNK ratio; (**C**) p-p38, p38, and p-p38/p38 ratio; and (**D**) p-MK2, MK2, and p-MK2/MK2 ratio. The results are expressed as mean ± SEM (* *p* < 0.05).

These observations prompted us to investigate the underlying mechanism of CUR in hepatic senescence. Activated MAPK pathways, including ERK1/2, JNK, and p38, are currently recognized as integral sensors for mitigating cellular senescence phenotypes [9]. We first examined the active form of ERK1/2 protein expression levels and found that there was no difference in the ratio of p-ERK1/2 to ERK1/2 in all four groups (Figure 4A). We then investigated CUR supplementation’s effect on JNK. The ratio of p-JNK to JNK protein expression levels was significantly decreased in the NCD+CUR group compared to the NCD group (*p* < 0.05), but not in the HFHSD-fed groups (Figure 4B). Lastly, we examined the ratio of p-p38 to p38 levels and found that the HFHSD+CUR-treated mice showed a decreased ratio compared to the HFHSD-treated mice (*p* = 0.0505; Figure 4C). To further confirm these results, we evaluated the expression levels of MK2, a downstream target of p38, which is mainly activated under stress and inflammatory stimuli [27]. Consistent with the p-p38 results, CUR treatment in the HFHSD group resulted in decreased protein expression levels of p-MK2 (*p* = 0.0761; Figure 2C), as well as a decreased ratio of p-MK2/MK2 (*p* = 0.0850; Figure 4D).

### 3.4. Dietary Curcumin Regulates Senescence-Associated Inflammatory Pathways in Aged Mice

**Figure 5:**
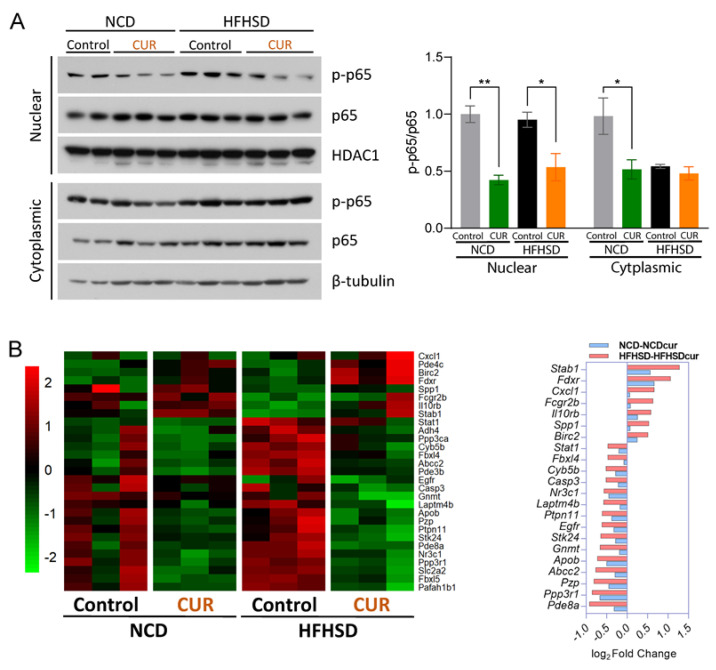
Curcumin downregulates inflammation-related pathways in aged mice. (**A**) Phosphorylated and total p65 nuclear and cytoplasmic protein expression levels in the liver tissue lysates. The results are expressed as mean ± SEM (* *p* < 0.05, ** *p* < 0.01). (**B**) Heatmap of inflammation-related mRNA expression measured using FPKM (*p* < 0.05 in HFHSD comparison; FPKM > 1.5) from the senescence pathway based on the GO term and KEGG analysis (red indicates a positive Z-score, and green indicates a negative Z-score) (left), and a bar chart of the genes with the most significant fold changes associated with the inflammation pathways based on the IPA presented with the Z-scores (red indicates activation and blue indicates suppression) (right).

It is well documented that the activation of MAPK pathways induces the downstream transcription factor, nuclear factor-κB (NF-κB), a crucial regulator of inflammation-associated gene expression and inflammatory responses [28]. Due to its canonical activation pathway via translocation from the cytoplasm to the nucleus, we fractionated proteins from the nuclear and cytoplasmic cellular compartments of the mouse liver tissues and measured the expression levels of phosphorylated and total p65 for both fractions. CUR supplementation significantly inhibited the ratio of nuclear p-p65 to p65 compared to the respective controls, the NCD (*p* < 0.01) and HFHSD (*p* < 0.05) groups (Figure 5A). Furthermore, the ratio of cytoplasmic p-p65 to p65 expression levels was significantly decreased by CUR supplementation in the NCD-fed mice (*p* < 0.05), suggesting the role of CUR in suppressing activated NF-κB as an effector of senescence in aged mice (Figure 5A).

**Figure 6:**
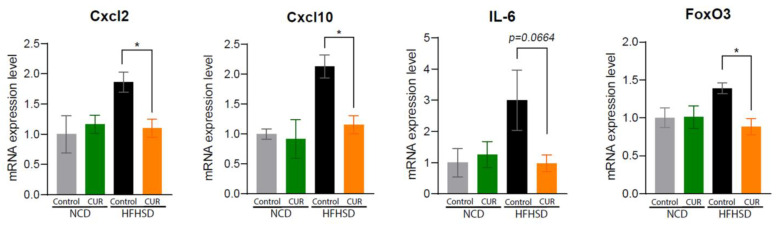
Curcumin suppresses senescence-associated secretory phenotypes in aged mice. mRNA expression levels of senescence-associated secretory phenotypes (SASPs): Cxcl2, Cxcl10, IL-6, and FoxO3. The results are expressed as mean ± SEM (* *p* < 0.05).

Considering the effect of CUR supplementation on the downregulation of NF-κB, we analyzed whether dietary CUR plays a role in the regulation of inflammatory genes. Thus, we selected inflammatory genes and analyzed their gene profiling using heatmap analysis. This analysis revealed that HFHSD+CUR led to the downregulation of genes involved in inflammation-related pathways (Figure 5B, left). Based on the Ingenuity Pathway Analysis (IPA), *Stab1*, *Fdxr*, *Ppp3r1*, and *Pde8a* showed the most significant fold changes in inflammation pathway-related gene expressions (Figure 5B, right). Next, we examined whether CUR supplementation negatively regulates SASPs because positive feedback loops between NF-κB and proinflammatory cytokines augment the amplification of SASPs [29]. Among the SASPs, CUR supplementation significantly downregulated the mRNA expression levels of chemokine (C-X-C motif) ligand 2 (Cxcl2), Cxcl10, and Forkhead box O3 (FoxO3) in the HFHSD group (*p* < 0.05; Figure 6). The mRNA expression level of interleukin 6 (IL-6) was also suppressed in the HFHSD+CUR group compared to the control HFHSD group (*p* = 0.0664; Figure 6).

### 3.5. Dietary Curcumin Prevents Body Weight Gain against Nutritional Challenges and Development of Insulin Resistance in Aged Mice

**Figure 7:**
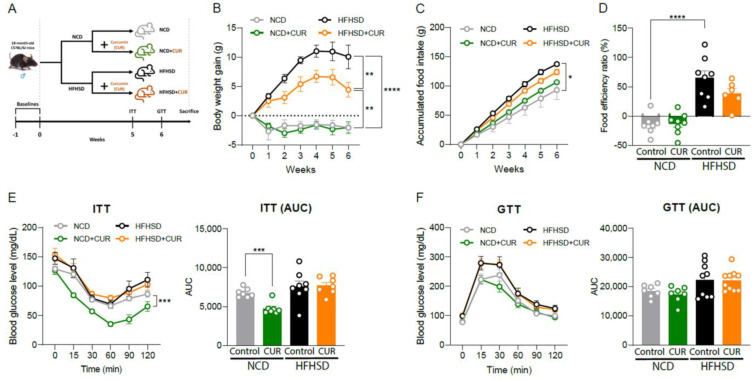
Mid-term curcumin administration mitigates obesity in diet-induced obese (DIO) aged mice. (**A**) 6-week animal experimental design. (**B**) Body weight gain (g), (**C**) cumulative food intake (g), and (**D**) food efficiency ratio (%) (*n* = 5–8 per group). (**E**) Insulin tolerance test (ITT) and (**F**) glucose tolerance test (GTT) (*n* = 7–10 per group). The results are expressed as mean ± SEM (* *p* < 0.05, ** *p* < 0.01, *** *p* < 0.005, and **** *p* < 0.001).

Previous studies have revealed that hepatic senescence is involved in impaired glucose and insulin homeostasis [30,31]. Since we found that CUR supplementation downregulates effectors of hepatic senescence, we investigated whether dietary CUR ameliorates age-associated symptoms, such as body weight gain against nutritional challenge and development of glucose intolerance and insulin resistance, in the NCD and HFHSD-fed aged mice (Figure 7A). Body weight gain in the HFHSD+CUR group was significantly suppressed compared to the HFHSD group (*p* < 0.01; Figure 7B), supporting the body weight-losing effect of CUR treatment after 15 weeks of intervention (Figure 1B). To further examine the reason for the prevention of body weight gain, we evaluated accumulated food intakes. Although the HFHSD-fed mice showed higher food intake than the NCD-fed mice, CUR supplementation did not make a significant difference in food intake within these groups (Figure 7C). We then calculated the food efficiency ratio by dividing body weight gains by cumulative food intakes (FER, %). The HFHSD group showed a higher percentage of FER than the NCD and NCD+CUR groups (*p* < 0.0001), but FER percentage was not changed by CUR intake in either the NCD or HFHSD groups (Figure 7D). These results indicate that CUR suppresses obesity due to HFHSD in aged mice without decreasing food intake. An ITT was performed at week 5, and CUR supplementation significantly improved insulin sensitivity in the NCD-fed mice (*p* < 0.005), but not in the HFHSD groups (Figure 7E). Meanwhile, a GTT at week 6 showed that there were no differences between CUR-treated and non-treated groups in both NCD and HFHSD dietary regimens (Figure 7F). Taken together, these results lend credence to the finding that CUR supplementation differently influences metabolic phenotypes depending on the dietary regimes of aged mice.

## 4. Discussion

CUR is renowned for its health benefits on age-related functional decline due to its anti-inflammatory, antiviral, anti-carcinogenic, and antioxidant effects [11,20,22,25]. Our recent study reported that dietary CUR also alters hepatic gene expression profiling and preserves insulin homeostasis in aged mice [11]. However, the underlying mechanism of how CUR supplementation regulates hepatic senescence is not fully understood.

In the current study, we found that CUR supplementation significantly downregulated 20 and 74 senescence-associated genes in the NCD and HFHSD groups, respectively. Of note, dietary CUR regulated senescence-related inflammatory genes including SASPs. These findings suggest that dietary CUR contributes to the inhibition of hepatic senescence by suppressing inflammatory gene expression.

The regulatory roles of MAPKs in senescent traits, including cell growth suppression, cell death resistance, and SASP regulations [9,32,33], emphasize the importance of targeting the MAPK pathways to eliminate senescent cells. Here, we observed that CUR supplementation was involved in the MAPK pathways in aged and nutritionally challenged aged mice. We found that CUR supplementation in the NCD groups led to a marked suppression of JNK based on the decreased protein expression ratio of p-JNK to JNK. It has been reported that the activation of JNK induces serine kinase phosphorylation of insulin receptor substrate (IRS)-1, which may block insulin signaling and eventually cause insulin resistance [34]. Consistent with this evidence, our results demonstrated that CUR supplementation improved insulin tolerance in the NCD-fed aged mice. JNK also promotes the cyclic guanosine monophosphate (GMP)–adenosine monophosphate (AMP) synthase (cGAS)–stimulator of interferon gene (STING) pathway, which not only triggers the secretion of SASP components but also activates the canonical NF-κB signaling pathways (RelA-p50) [35,36]. Despite no significant change in the mRNA expression of SASPs between the NCD and NCD+CUR groups, we noted that CUR supplementation significantly inhibited the expression of the p65 subunit of NF-κB in the NCD-fed aged mice. Therefore, we suggest a protective role of CUR via inhibiting the JNK/NF-κB signaling pathway in aged mice under normal diet conditions.

Previous studies have suggested that curcumin induces hepatic stellate cell (HSC) senescence via the activation of peroxisome proliferator-activated receptor gamma (PPARγ)/p53 signaling to inhibit the development of hepatic fibrosis [37]. However, in our study, we showed that CUR supplementation attenuated hepatic senescence via the suppression of p38. This discrepancy might be derived from these reasons: 1) different types and ages of liver cells and 2) different roles of CUR dependent upon liver conditions. The previous study used non-parenchymal cells (HSCs) and activated HSCs, which were chemically induced in vivo and in vitro to mimic a damaged liver, whereas our current study provided dietary CUR to naturally aged mice and analyzed the whole liver or parenchymal cells (primary hepatocytes). It is well documented that p38 MAPK leads to phosphorylation of p53, resulting in apoptosis induction in models of DNA damage, such as non-alcoholic fatty liver disease (NAFLD), steatohepatitis (NASH), and fibrosis [38,39]. Thus, CUR-induced senescence in a damaged liver may prevent the progress of liver diseases. Although aging itself is associated with instability of liver homeostasis, it is not a pathological condition. Thus, p53 gene and protein expression levels in the livers from naturally aged mice were not increased by the CUR supplementation (Supplementary Figure S2A,B). Conversely, the livers in the aged mice with nutritional challenges were similar to pathophysiological conditions with upregulated p53 gene expression levels (Supplementary Figure S2C,D). This suggests that dietary CUR delays hepatic senescence and provides health benefits with normal aging.

Generally, ERK1/2 and p38 simultaneously contribute to cellular senescence [9]. In this study, it is interesting to note that CUR did not modulate ERK1/2 activation in any of the four intervention groups, while CUR downregulated p38 protein expression in nutritionally challenged aged mice (Figure 4C). p38 regulates post-translational events for several downstream targets, including MK2 [40]. In our study, we observed the suppression of MK2 as well as p38 by CUR supplementation in the HFHSD group but not in the NCD group. Indeed, it has been reported that p38 activates NF-κB transcriptional activity, which, in turn, induces SASP production in senescent cells [41]. In the present study, CUR supplementation markedly downregulated the expression of p65, a key effector of transcription of pro-inflammatory cytokines among the NF-κB family members [41], followed by significant suppression of the mRNA expression levels of *Cxcl2*, *Cxcl10*, *IL-6*, and *FoxO3*. Additional evidence indicates that these inflammatory cytokines are highly secreted in obese and/or aged conditions [42,43]. Considering that dietary CUR significantly decreased the body weight gain in the HFHSD-fed mice, it could be postulated that the CUR-mediated suppression of the p38/NF-κB signaling pathway is dependent on the lack of weight gain, even though CUR does not influence insulin tolerance. These findings imply that CUR supplementation has the potential to be a senolytic agent that ameliorates metabolic dysfunctions.

Many researchers have already validated the pharmaceutical effects of CUR on obesity and glucose/insulin homeostasis through in vitro and in vivo studies [44,45,46,47]. Consistent with these studies, our previously published work proved that long-term (14–16 weeks) CUR supplementation enhanced insulin and glucose homeostasis in DIO-induced middle-aged and old mice [11,25]. In this study, we confirmed that a shorter period (6 weeks) of CUR supplementation also significantly reduced the body weight of the HFHSD-fed aged mice compared to the controls, and it restored insulin homeostasis in aged mice. However, with the 6-week administration, CUR did not affect glucose homeostasis in either the NCD or HFHSD groups, even though several previous studies have reported CUR ameliorates glucose tolerance [46,48]. We cannot rule out the characteristics of CUR as a fat-soluble compound with low bioavailability and the possibility of CUR oxidation within an animal diet [49], which might lead to the lower efficacy of CUR. Nevertheless, these data demonstrate that mid-term CUR administration has a therapeutic effect on obesity traits.

There are some limitations in the current study. We primarily targeted the whole liver and hepatocytes to investigate metabolic aging [50]. However, as a complex metabolic organ, the liver consists of various types of cells, such as HSC, Kupffer cells, and endothelial sinusoidal cells, which are all differently impacted by aging. Therefore, it may require further investigation to address CUR’s effects on hepatic senescence in other liver cell types with aging. Since CUR has low oral bioavailability, a dose–effect study examining plasma CUR levels would also be worthwhile. It is possible that other CUR formulations with increased solubility might also lead to greater beneficial effects. In addition, we conducted animal studies for about 1–3 months, but it is still unclear whether CUR intake shows preventive effects on hepatic cellular senescence with administrations longer than 6–12 months. Since several studies have proved that CUR prolongs lifespan in animal models [51,52], longer dietary interventions to examine health span expansion by CUR would be valuable.

## 5. Conclusions

Collectively, dietary CUR supplementation is likely to play a role in alleviating age-related hepatic senescence in aging itself and its combination with dietary challenges. The available data are convincing that CUR suppresses hepatic senescence via the downregulation of the JNK/NF-κB and p38/NF-κB pathways in normal and obese aged conditions, respectively. This is the first study to report CUR’s effects on hepatic cellular senescence and its health benefits as an aspect of metabolic homeostasis. Thus, CUR is a potent, natural therapeutic agent that acts in a multifaceted manner to protect against age-associated metabolic disorders. In summary, the results presented in this study suggest that CUR could function as a novel senolytic compound by suppressing hepatic cellular senescence.

## Data Availability

The datasets used and/or analyzed during the current study are available from the corresponding author upon reasonable request.

## Supplementary Materials

The following supporting information can be downloaded at https://www.mdpi.com/article/10.3390/antiox12061165/s1. Figure S1: Heatmaps of cellular senescence-related gene expressions: (A) Gene Ontology (GO; GO:0090398) and (B) KEGG pathway (hsa04218). Figure S2: Effect of curcumin on protein and gene expression of p53 in mouse primary hepatocytes. Curcumin (CUR, 10 μM) or palmitic acid (PA, 100 μM) were treated solely or together for 24 h. Table S1: Oligonucleotides for the real-time RT-PCR used in this study. Table S2: Abbreviations mentioned in this article. File S1: Original images for the Western blots.
