# Supplementary material for: Dietary Curcumin Attenuates Hepatic Cellular Senescence by Suppressing the MAPK/NF-κB Signaling Pathway in Aged Mice
Source: Antioxidants (Basel). 2023 May 27;12(6):1165. doi: 10.3390/antiox12061165 (PMC10294785; doi:10.3390/antiox12061165)
Supplement: Supplementary file 1 [file antioxidants-12-01165-s001.zip › antioxidants-2368467-supplementary.pdf]

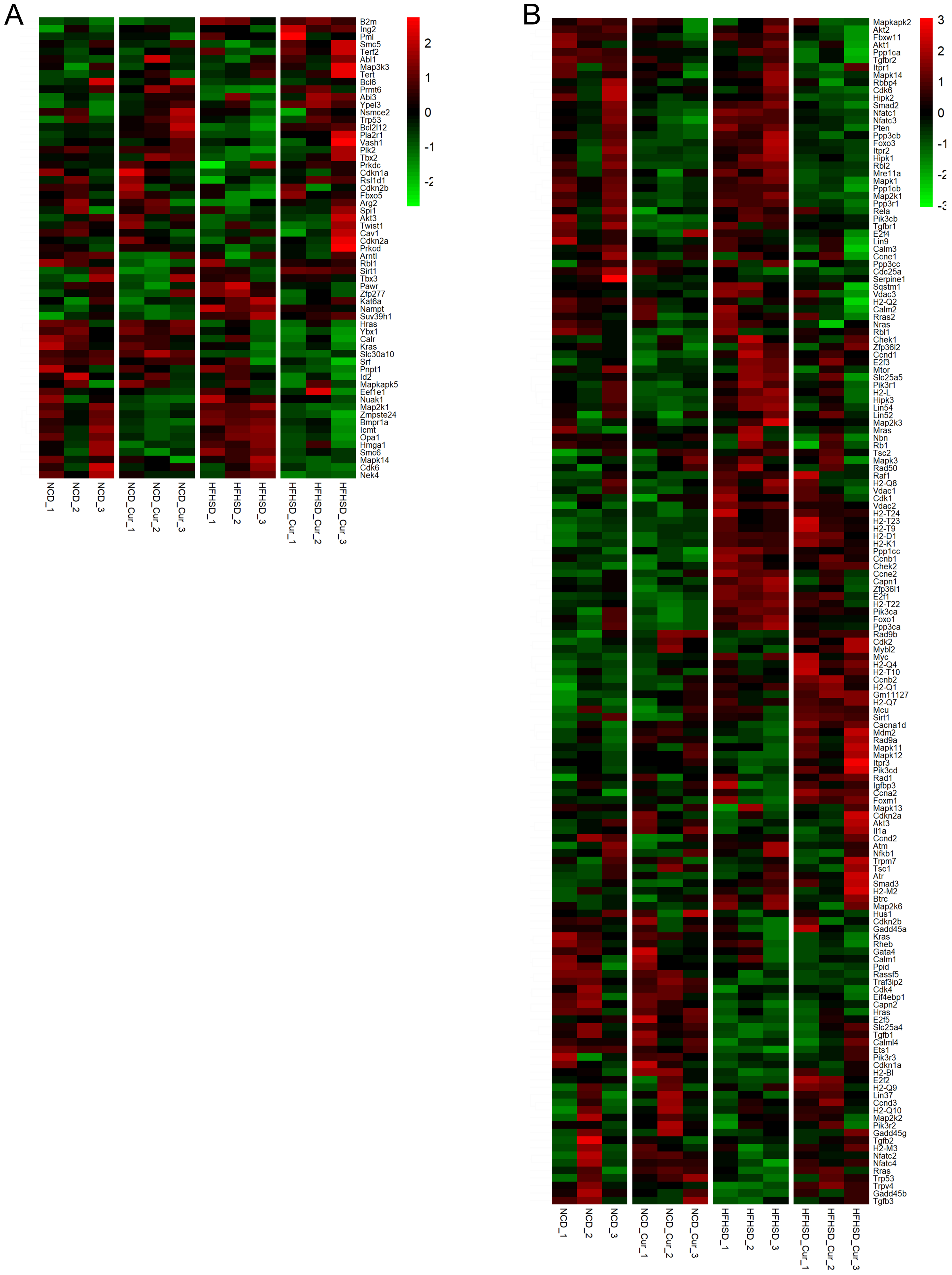

Figure S1. Lee et al.

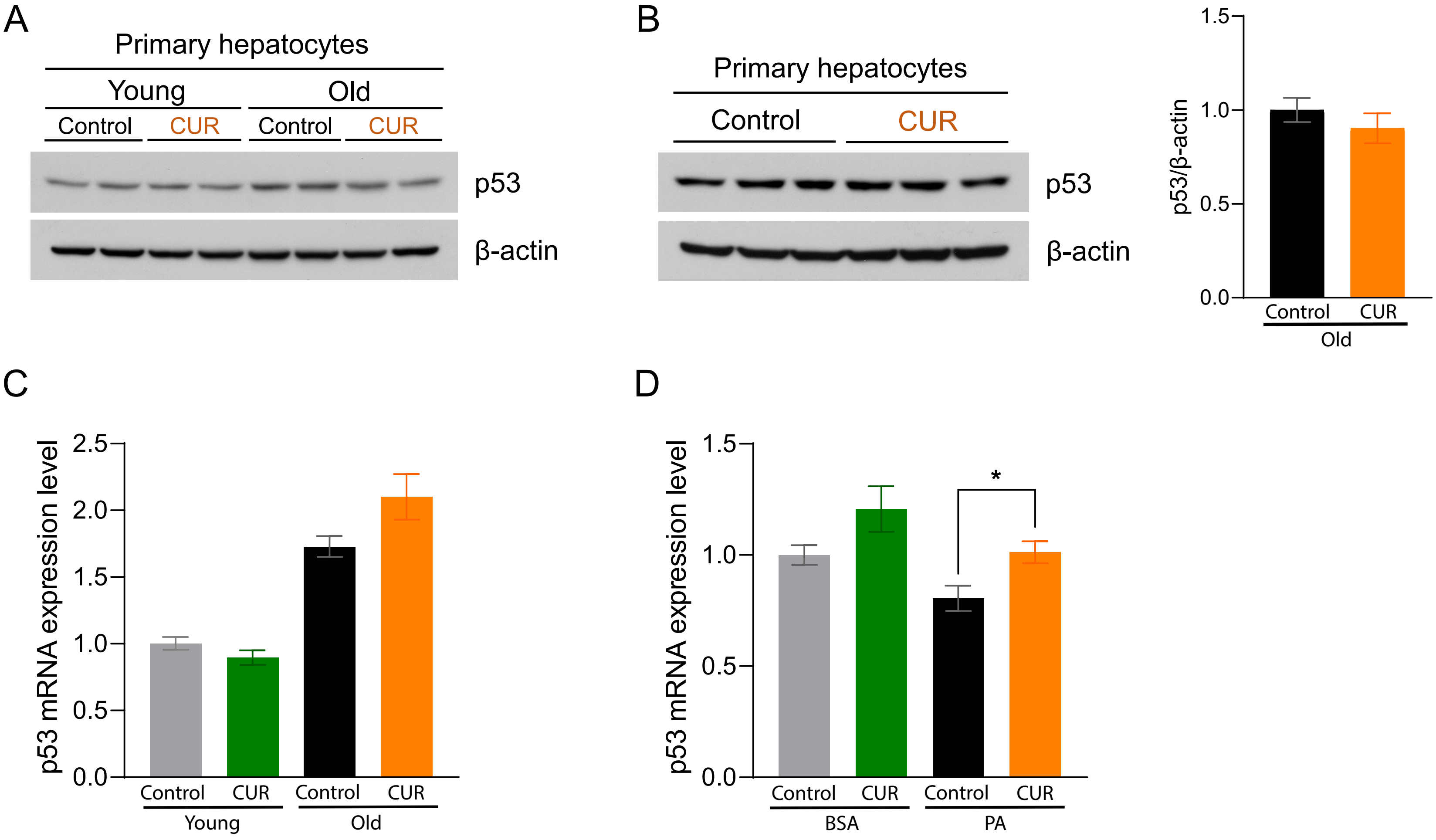

Figure S2. Lee et al.

Table S1. Oligonucleotides for *real-time* RT-PCR used in this study.

Origin	Target	5' – 3'	Sequence	Gene ID
Mus musculus	GPx1	Forward<br>Reverse	5'-CGGTTTCCCGTGCAATC-3'<br>5'-GAGGGAATTCAGAATCTCTTCAT-3'	14775
	GPx2	Forward<br>Reverse	5'-TGTGGCGTCACTCTGAGGAA-3'<br>5'-GAAGCGACATTGCAGCTCAT-3'	14776
	GPx3	Forward<br>Reverse	5'-AACTCGGAGATACTCCCCAGTCT-3'<br>5'-GCTGGAAATTAGGCACAAAGC-3'	14778
	GPx4	Forward<br>Reverse	5'-ACCCACTGTGGAAATGGATGA-3'<br>5'-CTCTATCACCTGGGGCTCCTC-3'	625249
	HMOX1	Forward<br>Reverse	5'-TCAGGTGTCCAGAGAAGGCTTT-3'<br>5'-TCTTCCAGGGCCGTGTAGAT -3'	15368
	Txn1	Forward<br>Reverse	5'-GTGGTGTTCCTTGAAGTGGAT-3'<br>5'-GGCATGCATTTGACTTCACAGT-3'	22166
	p53	Forward<br>Reverse	5'-GCTTCTCCGAAGACTGGATGA-3'<br>5'-GAGTTTCCATAAGCCTGAAAATGTC -3'	22059
	Cxcl2	Forward<br>Reverse	5'-CCCAGACAGAAGTCATAGCCAC-3'<br>5'-TGGTTCTTCCGTTGAGGGAC-3'	20310
	Cxcl10	Forward<br>Reverse	5'-CGCTGCAACTGCATCCATATCG-3'<br>5'-CCGGATTCAGACATCTCTGCTC-3'	15945
	IL-6	Forward<br>Reverse	5'-TGAGAAAAGAGTTGTGCAATGG-3'<br>5'-GGTACTCCAGAAGACCAGAGG-3'	16193
	FoxO3	Forward<br>Reverse	5'-CTCCATCCGGCACAACCT-3'<br>5'-TTGCCCGTCCCTTCATTC-3'	56484
	β-actin	Forward<br>Reverse	5'-TACCACCATGTACCCAGGCA-3'<br>5'-CTCAGGAGGAGCAATGATCCTGAT-3'	11461
	18S Ribosomal RNA	Forward<br>Reverse	5'-CGATCCGAGGGCCTCATCA-3'<br>5'-AGTCCCTGCCCTTTGTACACA-3'	19791

GPx, glutathione peroxidase; HMOX, heme oxygenase; Txn, thioredoxin; Cxcl, chemokine (C-X-C motif) ligand; IL, interleukin; FoxO3, Forkhead box O3.

Table S2. Abbreviations mentioned in this article.

Abbreviation	Meaning
AMP	Adenosine monophosphate
ANOVA	Analysis of variance
AUC	Area under the curve
BCA	Bicinchoninic acid
CUR	Curcumin
Cxcl	Chemokine (C-X-C motif) ligand
DEG	Differentially expressed genes
DIO	Diet-induced obesity
ERK	Extracellular signal-regulated kinase
FER	Food efficiency ratio
FOX	Forkhead box
FPKM	Fragments per kilobase of transcript per million mapped reads
GEO	Gene Expression Omnibus
GMP	Guanosine monophosphate
GO	Gene Ontology
GPx	Glutathione peroxidase
GTT	Glucose tolerance test
HDAC1	Histone deacetylase 1
HFHSD	High-fat high-sugar diet
HMOX1	Heme oxygenase 1
IL	Interleukin
IPA	Ingenuity Pathway Analysis
IRS	Insulin receptor substrate
ITT	Insulin tolerance test
JNK	c-Jun N-terminal kinase
KEGG	Kyoto Encyclopedia of Genes and Genomes
MAPK	Mitogen-activated protein kinase
MGI	Mouse Genome Informatics
MK	MAP kinase-activated protein kinase
NCD	Normal chow diet
NF-κB	Nuclear factor kappa B
PVDF	polyvinylidene fluoride
RT-PCR	Reverse-transcription polymerase chain reaction
SASP	Senescence-associated secretory phenotype
SDS-PAGE	Sodium dodecyl sulfate–polyacrylamide gel electrophoresis
SEM	Standard error of the mean
STING	Stimulator of interferon genes
Txn	Thioredoxin
